# Bilateral lotus petal flap reconstruction for perianal Paget’s disease

**DOI:** 10.1007/s10151-020-02283-w

**Published:** 2020-06-30

**Authors:** Ugo Grossi, Giulio Aniello Santoro, Elisa Antoniazzi, Francesco Dell’Antonia, Enrico Busato, Giorgio Berna, Giacomo Zanus

**Affiliations:** 1 Tertiary Referral Pelvic Floor and Incontinence Centre, DISCOG, Treviso Regional Hospital, University of Padua, Treviso, Italy; 2 National Bowel Research Centre, Queen Mary University of London, London, UK; 3 Department of Plastic and Reconstructive Surgery, Treviso Regional Hospital, Treviso, Italy; 4 Department of Obstetrics and Gynecology, Treviso Regional Hospital, Treviso, Italy

**Fig. 1:**
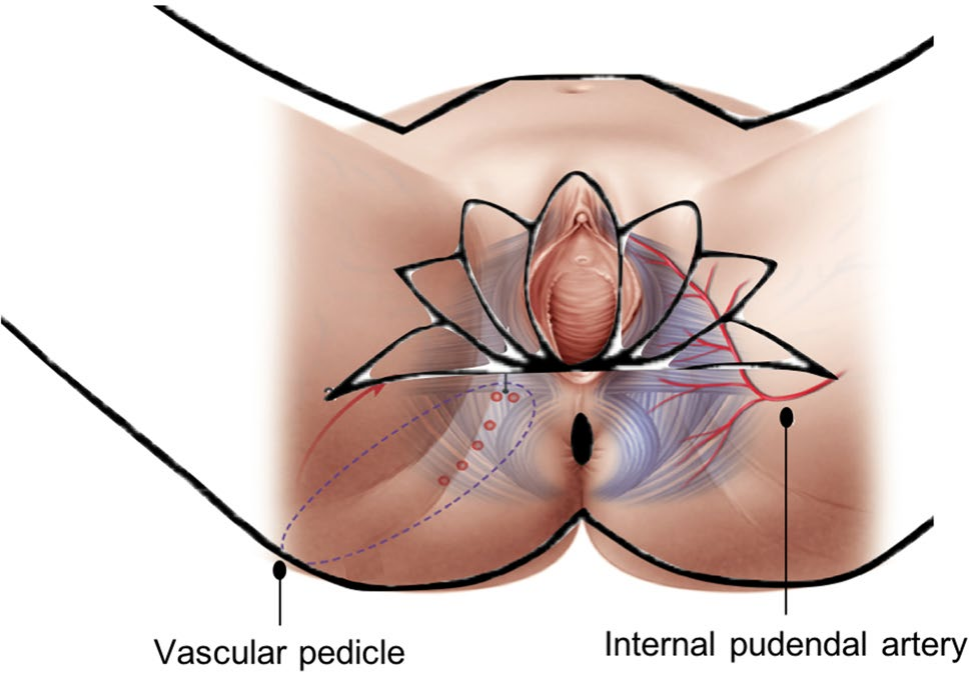
Schematic representation of the lotus petal flap reconstruction

**Fig. 2:**
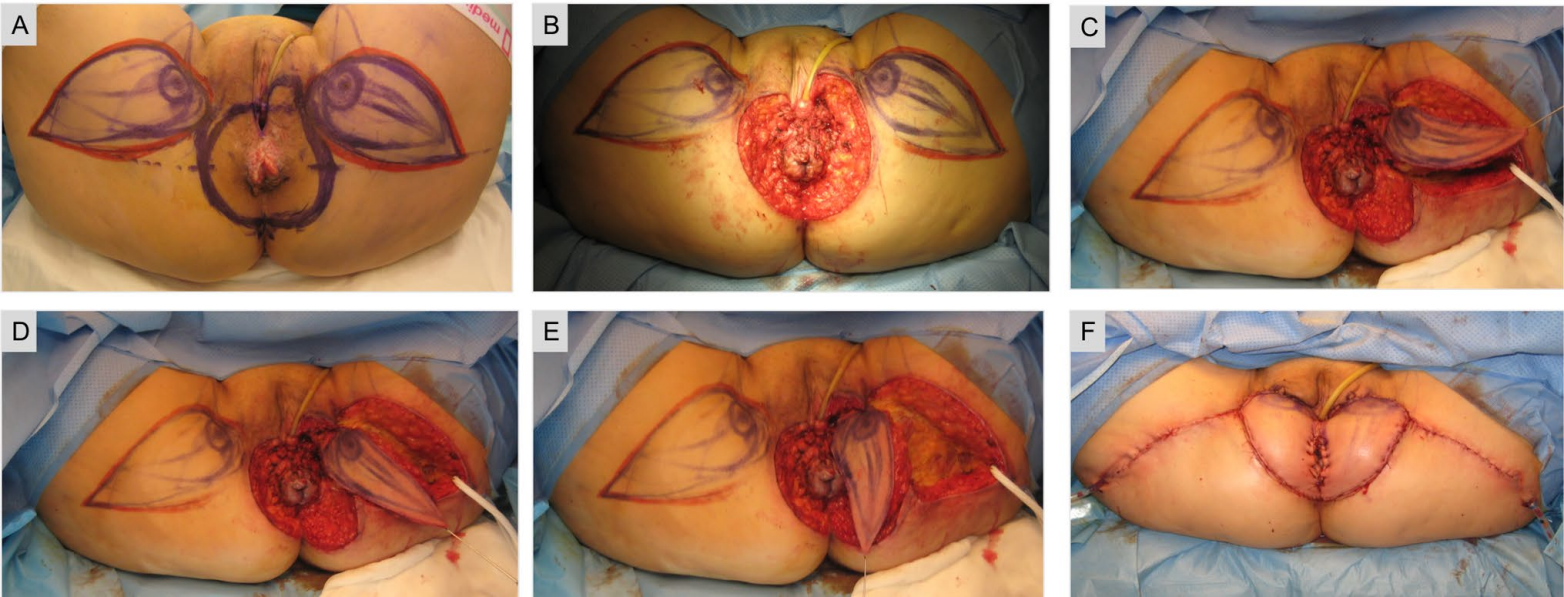
Flap planning was performed with handheld Doppler ultrasound to detect perforators of the internal pudendal artery. The excision included all visible lesions in the perianal skin and the posterior part of the vulva, with a 2 cm margin of healthy tissue (**a**). The outer 1.5 cm of the urethra were mobilized and the transverse perineal muscles exposed, along with the external anal sphincter (**b**). Two fasciocutaneous lotus petal flaps were centred over the medial thigh crease bilaterally. They were elevated in an posterior-to-anterior direction and posteriorly rotated with a 90° angle to cover the perianal defect. A Foley urinary catheter was left in situ for a total of 7 days (**c**–**e**). Immediate postoperative result after flap in-setting and primary closure of the donor site (**f**)

**Fig. 3:**
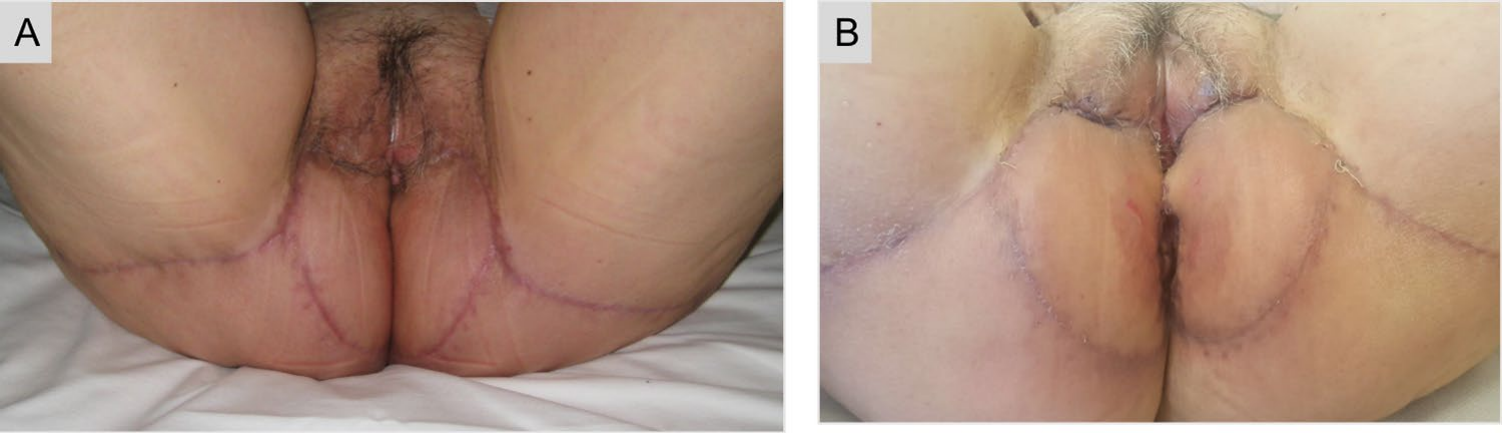
The patient was continent to faeces and urine, with no signs of wound infection, breakdown or haematoma at 4 (**a**) and 12 weeks (**b**) postoperatively

A 65-year-old female patient with a history of intermittent pruritus for the past 2 years, presented with erythematous plaques with white scaling in the perineal, vulvar and perianal regions. Skin biopsy was consistent with Paget’s disease [1]. Given the extension of the lesions, a wide local excision and bilateral lotus petal flap reconstruction was carried out by an institutional multidisciplinary panel (Figs. 1, 2) [2, 3]. The patient recovered uneventfully. The surgical wounds healed completely without flap compromise and there was normal sphincter function at the 3-month follow-up (Fig. 3).
